# Right tool for the right job in the right way: robotic, transanal, or laparoscopic approach for rectal cancer

**DOI:** 10.1093/bjsopen/zrae069

**Published:** 2024-06-13

**Authors:** Deborah S Keller

**Affiliations:** Division of Digestive Surgery, University of Strasbourg, Strasbourg, France

A high-quality total mesorectal excision (TME) is independently predictive of reducing local recurrence in rectal cancer surgery^1,2^. The authors performed a retrospective propensity score-matched comparison across three minimally invasive approaches (laparoscopic, robotic and transanal (TaTME)) on intraoperative, postoperative, quality and survival outcomes to determine the best TME approach^3^. They reviewed patients with rectal cancer within 12 cm of the anal verge from eight expert centres in the European MRI and Rectal Cancer Surgery III (EuMaRCS) database over a decade. The results demonstrated that laparoscopy was associated with higher conversion to open, higher postoperative complications and lower rates of stoma closure. Concerns were found with TaTME, where only 58% had more than 12 harvested lymph nodes and higher rates of Grade C anastomotic leakage were found (4.1% *versus* 0.7% robotic and 0% laparoscopic). The authors concluded that robotic and TaTME approaches were the best platforms for performing a TME.

Here I push back, as the best approach for performing a TME varies with each patient and multiple variables require consideration to make the best decision. The ideal approach depends on patient factors and preference; the disease and its response to neoadjuvant therapy; the case environment and platforms available; and the surgeon performing the operation. The safety, feasibility, superior postoperative outcomes and similar oncologic results over open surgery have been proven for all approaches with experienced surgeons. Laparoscopy is well established, with platforms widely available and most colorectal surgeons proficiently trained in the technique, but challenges are encountered from the rigid instrumentation, ergonomic strain, visibility and exposure in the deep pelvis. Laparoscopy is not the easiest platform in men with a narrow pelvis, obese patients and those with bulky tumours. Robotic-assisted platforms overcome the technical and ergonomic challenges of laparoscopy through wristed instruments with multiple degrees of freedom, additional arms for retraction and exposure, and 3D high-definition magnified views, which can translate to a more precise dissection at any level of the rectum. This has resulted in lower conversion rates than laparoscopy and shorter times to recover bowel function. The robotic platform may be ideal for any TME procedure, however, there are high costs for acquisition, service contracts, equipment, and rigorous training of the surgeon and team. TaTME is ideal for low rectal cancers and obese patients, as it mitigates against limitations to achieve optimal exposure and reach the pelvic floor. By starting transanally, the most challenging part of the operation is performed first with certainty regarding the distal margin. For surgeons without access or appropriate experience with robotics, combining the transanal and laparoscopic abdominal approach is an effective solution for low rectal cancers. However, there is a distinct learning curve for pattern recognition and anatomic landmarks in the bottoms-up view that requires structured training, proctoring and stringent case selection to avoid serious injury.

In the present work, insufficient information was provided to decide on the best approach. First, the wrong timeframe for assessment of patient and tumour variables was used; all were either before any neoadjuvant treatment or based on the surgical pathology. Decision-making for the procedure (and approach) is most accurate 6–8 weeks after traditional neoadjuvant chemoradiation or 10–12 weeks after total neoadjuvant therapy^4,5^. This allows assessment of treatment response and stratification of patients likely to achieve a complete clinical response from those exhibiting tumour progression or poor response. There was no information on the cancer from examination or imaging, which is critical for selecting the best approach. On digital rectal examination and endoscopy during this postradiation assessment, the size, configuration, circumference of the lumen encompassed, and distance from the anal verge and anorectal ring are most accurately assessed. The postradiation T2-weighted MRI is also instrumental for tumour regression, the relationship of the cancer to the mesorectal fascia, involvement of surrounding structures and poor prognostic features which could impact the treatment plan^6^. None of these variables were presented for assessment. In addition, there was no information on each surgeon’s individual experience. The authors noted, ‘The surgical procedures…were all performed by experienced surgeons in university hospitals’; however, this does not clarify that all surgeons participating were proficient and had access to all platforms analysed. This is compulsory information to reduce selection bias. Parameters the authors presented that could be helpful, such as ‘location’ were too vague to be actionable. Is this the location of the distal margin to the anal verge? Or the anorectal ring? And is the value still accurate after the patient received chemoradiotherapy? The details matter. Finally, interpretation of results for several outcomes warrants review. The authors reported no differences in the incidence of intraoperative complications across platforms, from a *P* value of 0.055, but the rate in laparoscopic cases was more than double robotic and TaTME rates (10.8% *versus* 4.7%). There were no details on what constituted an intraoperative complication, or the relationship of intraoperative complications to major postoperative complications, though this discrepancy is clinically significant to surgeons performing a TME. Furthermore, when discussing dreaded anastomotic complications, the authors highlight benefits for robotics in International Study Group of Rectal Cancer Grade A leaks; however, these usually do not change clinical management; emphasizing the statistically significantly higher rate of Grade C anastomotic leakage in the transanal group may be more relevant for surgeons to consider when choosing a platform.

In summary, this comparative work on selecting the best TME platform is an important addition to the literature. However, there are issues with several variables selected for analysis, the generalizations and interpretation of results. Several critical points for the individual surgeon to consider when selecting an approach are highlighted here. Not all patients are the same. Not all cancers are the same. Not all surgeons have the same experience and access to all platforms. And not all platforms serve the same benefit. The best approach for the TME is the one that allows the individual surgeon to perform the highest quality procedure in the least invasive fashion on the patient and cancer at hand.
